# Risk factors for the development of nosocomial pneumonia and mortality on intensive care units: application of competing risks models

**DOI:** 10.1186/cc6852

**Published:** 2008-04-02

**Authors:** Martin Wolkewitz, Ralf Peter Vonberg, Hajo Grundmann, Jan Beyersmann, Petra Gastmeier, Sina Bärwolff, Christine Geffers, Michael Behnke, Henning Rüden, Martin Schumacher

**Affiliations:** 1 Institute of Medical Biometry and Medical Informatics, University Medical Center Freiburg, Freiburg, Germany; 2 Institute for Medical Microbiology and Hospital Epidemiology, Medical School Hannover, Hannover, Germany; 3 European Antimicrobial Resistance Surveillance System, National Institute for Public Health and the Environment, Bilthoven, The Netherlands; 4 Institute of Hygiene and Environmental Medicine, Charité – University Medicine, Berlin, Germany

## Abstract

**Introduction:**

Pneumonia is a very common nosocomial infection in intensive care units (ICUs). Many studies have investigated risk factors for the development of infection and its consequences. However, the evaluation in most of theses studies disregards the fact that there are additional competing events, such as discharge or death.

**Methods:**

A prospective cohort study was conducted over 18 months in five intensive care units at one university hospital. All patients that were admitted for at least 2 days were included, and surveillance of nosocomial pneumonia was conducted. Various potential risk factors (baseline- and time-dependent) were evaluated in two competing risks models: the acquisition of nosocomial pneumonia and discharge (dead or alive; model 1) and for the risk of death in the ICU and discharge alive (model 2).

**Results:**

Patients from 1,876 admissions were included. A total of 158 patients developed nosocomial pneumonia. The main risk factors for nosocomial pneumonia in the multivariate analysis in model 1 were: elective surgery (cause-specific hazard ratio = 1.95; 95% CI 1.33 to 2.85) or emergency surgery (1.59; 95% CI 1.10 to 2.28) prior to ICU admission, usage of a nasogastric tube (3.04; 95% CI 1.25 to 7.37) and mechanical ventilation (5.90; 95% CI 2.47 to 14.09). Nosocomial pneumonia prolonged the length of ICU stay but was not directly associated with a fatal outcome (p = 0.55).

**Conclusion:**

More studies using competing risk models, which provide more accurate data compared to naive survival curves or logistic models, should be carried out to verify the impact of risk factors and patient characteristics for the acquisition of nosocomial infections and infection-associated mortality.

## Introduction

Nosocomial pneumonia (NP) is the most commonly reported infection in intensive care units (ICUs), especially in mechanically ventilated patients with an incidence of about 15 infections per 1,000 ventilation days [1]. This infection is associated with a significantly increased length of hospital stay and may have a considerable impact on morbidity and mortality [2].

Endpoints, possible risk factors for the acquisition of NP and the clinical outcome after the infection has occurred have been addressed in numerous studies. However, many of these studies did not take into account the fact that there are other possible endpoints competing with the event of interest [3,4]. For example 'death' or 'discharge' are competing events for the onset of infection. A competing risks methodology allows for a better understanding of why NP increases mortality. Unlike logistic regression, it allows modelling of the time-dependency of certain procedures (for example intubation), thereby avoiding biased results. For this, multi-state models are a more accurate approach in order to consider competing events [5,6]. We present here the results of a competing risks analysis to address two major objectives: (1) to identify potential risk factors for NP in ICUs, considering discharge (dead or alive without prior NP) as the competing event, and (2) to investigate several risk factors, including blood stream infection, NP and other lower respiratory tract infections as time-dependent risks, for mortality in ICU patients with discharge (alive) as the competing endpoint.

## Materials and methods

### Patients and infections

The presenr study was conducted in five ICUs (one medical, one surgical, one neurosurgical and two interdisciplinary) at one German university hospital from February 2000 to July 2001 (a total study period of 18 months). All patients with a duration of ICU stay of at least 2 days were enrolled. Prospective surveillance of nosocomial infections was performed by trained staff of the German Nosocomial Infection Surveillance System (KISS) [7] using the standardized US Centers for Disease Control and Prevention (CDC) definitions for NP [8]. The method of surveillance remained unchanged over the study period. As all investigations represented routine diagnostic procedures, the Institutional Board on the Ethics of Clinical Studies waived the need for informed consent. Further details on the setting of the study are described elsewhere [9,10].

### Analysis of risk factors for the acquisition of NP (model 1)

**Figure 1:**
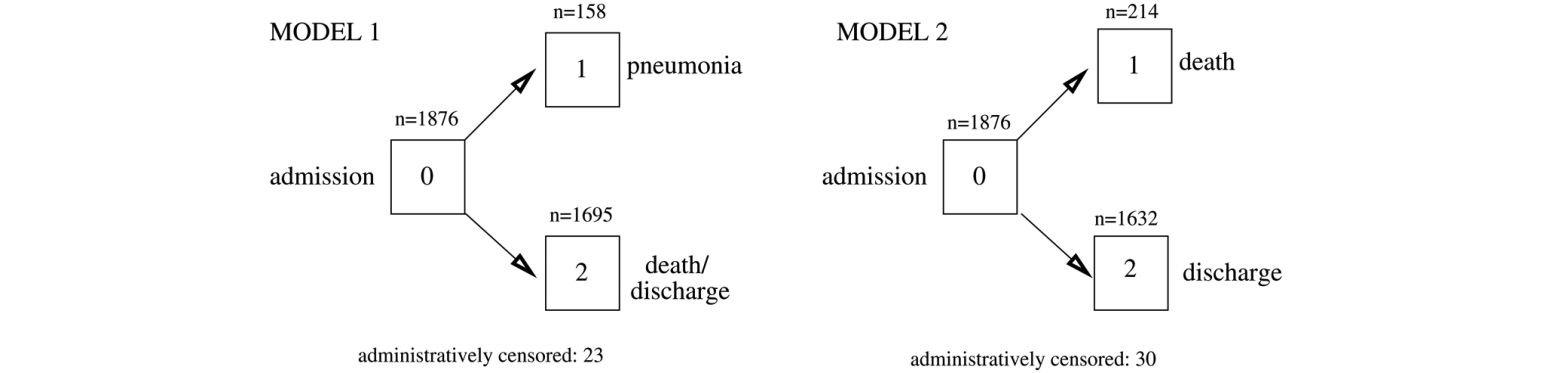
Competing endpoints in model 1 and model 2.

In model 1, we studied risk factors for NP acquisition as well as the competing risk 'discharge (dead or alive without prior NP)' (Figure 1). After admission to the ICU (event 0) the patient may (event 1) or may not (event 2) acquire NP. The impact of the following baseline risk factors were investigated: age, gender, simplified acute physiology score (SAPS) II, intubation at ICU admission, infection present already at the time point of ICU admission (pneumonia, urinary tract infection and other infections), hospitalization prior to ICU admission, elective or emergency surgery before ICU admission (for example, head trauma, multiple trauma, vascular surgery and neurosurgery), underlying diseases (cardial/pulmonal, gastrointestinal, neurological, and metabolic/renal) and other underlying diseases (including sepsis, malignancies or alcoholism). The impact of the following time-dependent risk factors were investigated as time-dependent covariates (which start with value = 0 and may increase to 1): ventilation, chest drainage, colostomy, enterostomy, jejunostomy, nasogastric tube and urinary catheter. Age and SAPS II score were included in the model as continuous variables; all other factors were binary variables only.

### Analysis of risk factors for mortality (model 2)

In model 2 we studied competing risks for mortality and discharge (Figure 1). After admission to the ICU (event 0) the patient may either die during their ICU stay (event 1) or be discharged from the ICU (event 2). Here, we are mainly interested in NP as a time-dependent risk factor for death in the ICU. The same baseline and time-dependent risk factors as described for model 1 were also applied in model 2. We also checked for lower respiratory tract (LRT) infections other than pneumonia on admission as baseline, and for nosocomial LRT and nosocomial blood stream infection as time-dependent variables.

For both models 1 and 2 a competing risk analysis was performed using cause-specific hazards [11,12]. This analysis follows separate Cox models for each event assuming proportional hazards. In such competing risks analyses with two endpoints, it is possible to interpret both cause-specific hazard ratios (CSHRs) simultaneously for each risk factor. Cumulative incidence functions have been displayed for each endpoint. The proportional hazard assumptions were assessed by study of the graphs of the Schoenfeld's residuals; this technique is especially suitable for time-dependent covariates [13]. The correlation matrices of each Cox model were considered in order to check whether there are correlations among the risk factors, respectively. Risk factors with a p value ≤ 0.157 for at least one of the CSHRs from the univariate analysis were included in a consecutive multivariate analysis. This benchmark corresponds to the well established Akaike information criterion for model selection [14]. A p value ≤ 0.05 was considered statistically significant. For all analyses the R 2.4.1 software was used (R Foundation, Vienna, Austria), especially the R functions *coxph, cuminc *and *cox.zph*, from the *survival *and *cmprsk *libraries.

Additional data file 1 contains information on the required data format and SAS and R calculations for cause-specific hazard ratios in a competing risks analysis with time-dependent covariates represented.

## Results

### Patients and infections

A total of 7,269 patients were admitted to the ICUs (35,817 patient days) during the study period; of those, 1,876 admissions (28,498 patient days) required treatment of ≥ 48 h. Only those patients were included in this study. In all, 158 (8.4%) of the included patients developed NP; 132 of these (83.5% of all NP) were ventilator-associated NP (incidence of 8.5 per 1,000 ventilator days) and 33 of these (20.9% of all NP cases) died in the ICU. Overall, in 214 of the 1,876 admissions (11.4%) the patient died in the ICU. More details of risk factors and outcomes are shown in Table 1.

**Table 1:** Descriptive results of all risk factors and outcomes for all 1,876 admissions

**Variables**		
Continuous:	Mean	SD

Age	60.0	18.4
SAPS II	35.2	18.7
		
Binary:	Number	Percentaqe

Female gender	764	40.72
Intubation on admission	848	45.20
Pneumonia on admission	220	11.73
LRT on admission	24	1.28
Urinary tract infection on admission	42	2.24
Other infections on admission	139	7.41
Hospitalization before admission	1,334	71.11
Surgical patients	433	23.08
Elective surgery before admission	883	47.07
Emergency surgery before admission	456	24.31
Cardial/pulmonary underlying disease	653	34.81
Neurological underlying disease	370	19.72
Metabolic/renal underlying disease	180	9.59
Other underlying disease	180	9.59
		
Time-dependent events (binary)	Number of events	Time (days) to event among those with event (Q25, median, Q75)

Discharge from ICU (alive)	1,632	(5,8,17)
Death in the ICU	214	(7,13,27)
Nosocomial pneumonia	158	(5,8,14)
Nosocomial blood stream infection	35	(7,13,26)
Nosocomial LRT	33	(5,6,10)
Ventilation	1,041	(1,1,1)
Chest drainage	366	(1,1,1)
Colostomy	44	(1,1,1)
Enterostomy	59	(1,1,1)
Jejunostomy	23	(1,1,10)
Nasogastric tube	1,263	(1,1,1)
Urinary catheter	1,608	(1,1,1)

ICU, intensive care unit; LRT, lower respiratory tract infection (other than pneumonia); Q, quartile; SAPS, simplified acute physiology score.

### Analysis of risk factors for the acquisition of nosocomial pneumonia (model 1)

Detailed information on the CSHRs of baseline and time-dependent risk factors of model 1 are shown in Table 2. According to this model, significant risk factors for the acquisition of NP in our patient population were (1) pneumonia at admission (CSHR = 0.02), whereas this risk factor also had a reducing effect on the competing event discharge (CSHR = 0.66), (2) undergoing elective surgery prior to ICU admission (CSHR = 1.95), and this effect was accentuated since the CSHR was reduced for discharge (CSHR = 0.54), (3) undergoing emergency surgery prior to ICU admission (CSHR = 1.59), with no significant effect on discharge (CSHR = 1.08), (4) use of a nasogastric tube (CSHR = 3.04), without effect on discharge (CSHR = 0.89), and (5) mechanical ventilation of the patient (CSHR = 5.90), which also significantly reduced the CSHR for discharge from the ICU (CSHR = 0.53; 95% CI 0.45 to 0.62).

**Table 2:** Multivariate analysis of cause-specific hazard ratios for the acquisition of nosocomial pneumonia (model 1)

	**Possible endpoints (competing risks)**					
**Risk factor**						
	**Nosocomial pneumonia**			**Discharge (dead or alive)**		
	
	**CSHR**	**95% CI**	**p Value**	**CSHR**	**95% CI**	**p Value**
Baseline:						
Age (continuous variable)	1.01	1.00 to 1.02	0.18	1.00	1.00 to 1.01	0.01
Female gender	0.75	0.53 to 1.07	0.12	1.10	0.99 to 1.22	0.07
SAPS II (continuous variable)	1.00	0.98 to 1.01	0.42	0.98	0.98 to 0.99	< 0.01
Intubation on admission	0.89	0.71 to 1.13	0.35	1.05	0.96 to 1.14	0.32
Pneumonia on admission	0.02	0.00 to 0.12	< 0.01	0.66	0.56 to 0.77	< 0.01
Urinary tract infection on admission	1.86	0.60 to 5.82	0.28	0.81	0.56 to 1.18	0.28
Other infections on admission	1.08	0.59 to 1.98	0.79	0.72	0.59 to 0.89	< 0.01
Hospitalization before admission	0.73	0.50 to 1.05	0.09	0.91	0.81 to 1.02	0.10
Surgical patients	0.69	0.41 to 1.18	0.18	0.98	0.83 to 1.16	0.80
Elective surgery before admission	1.95	1.33 to 2.85	< 0.01	0.54	0.48 to 0.60	< 0.01
Emergency surgery before admission	1.59	1.10 to 2.28	0.01	1.08	0.95 to 1.23	0.25
Cardial/pulmonary underlying disease	1.32	0.86 to 2.04	0.20	0.84	0.73 to 0.97	0.02
Neurological underlying disease	1.25	0.78 to 2.00	0.36	0.94	0.81 to 1.09	0.41
Metabolic/renal underlying disease	0.76	0.35 to 1.65	0.48	0.80	0.65 to 0.99	0.04
Other underlying disease	1.49	0.83 to 2.66	0.18	1.00	0.81 to 1.24	1.00
Time-dependent:						
Ventilation	5.90	2.47 to 14.09	< 0.01	0.53	0.45 to 0.62	< 0.01
Chest drainage	1.00	0.68 to 1.46	0.99	0.75	0.65 to 0.86	< 0.01
Colostomy	4.29	0.36 to 50.64	0.25	0.69	0.28 to 1.72	0.42
Enterostomy	0.14	0.01 to 2.10	0.15	1.64	0.61 to 4.45	0.33
Jejunostomy	2.47	0.45 to 13.58	0.30	0.41	0.16 to 1.04	0.06
Nasogastric tube	3.04	1.25 to 7.37	0.01	0.89	0.76 to 1.03	0.12
Urinary catheter	1.53	0.49 to 4.81	0.46	0.76	0.65 to 0.90	< 0.01

CSHR, cause-specific hazard ratio; SAPS, simplified acute physiology score.

In addition to the analysis of model 1, we considered a model with three competing events: nosocomial pneumonia, discharge (alive) and death in the ICU. The CSHRs for pneumonia are the same as in model 1 with the combined competing event. However, the following risk factors had an opposite influence on discharge (alive) and death in the ICU: SAPS II, other infections on admission, surgical patients, metabolic/renal underlying disease and other underlying diseases. This is in line with the results for model 2.

### Cumulative incidence functions (CIF)(model 1)

**Figure 2:**
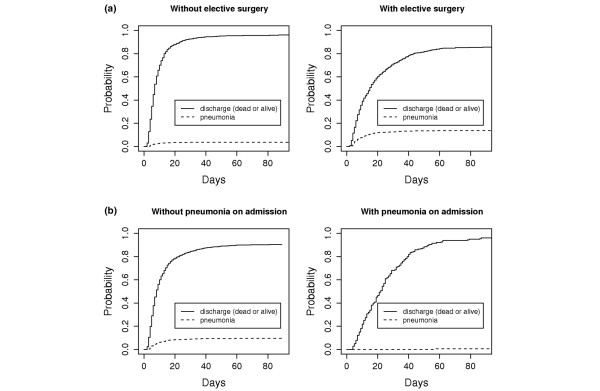
Cumulative incidence function for nosocomial pneumonia and discharge (dead or alive) (model 1). **(a) **In the two upper figures the risk factor 'elective surgery' is considered. **(b) **In the two lower figures the risk factor 'pneumonia on admission' is considered.

In addition to CSHR, cumulative incidence functions are suitable to illustrate the results of a competing risk analysis. This was exemplarily performed for the risk factors of elective surgery and pneumonia on admission. The CIF of pneumonia starts to increase at an earlier time point for patients with elective surgery, but later for the competing endpoint death/discharge (Figure 2a).

There is only a very low cause-specific risk to acquire nosocomial pneumonia if the patient already had pneumonia on admission (Figure 2b). Regarding discharge (dead or alive) as the endpoint, the cumulative incidence function of the patient group with pneumonia on admission is below the function of the group without until about 40 days in the ICU, but above afterwards.

### Analysis of risk factors for mortality (model 2)

Detailed information on the CSHRs of baseline and time-dependent risk factors of model 2 are shown in Table 3. The baseline variables of age, SAPS II and other underlying diseases significantly increased the CSHR for a fatal outcome. No nosocomial infection was significantly associated with the CSHR for death. However, patients with nosocomial pneumonia stay significantly longer in the ICU (CSHR = 0.59); a similar effect was seen for patients with nosocomial LRT (CSHR = 0.56). The CSHRs with regard to death in the ICU were not significant for these nosocomial infections.

**Table 3:** Multivariate analysis of cause-specific hazard ratios for mortality on intensive care units (model 2)

	**Possible endpoints (competing risks)**					
	
**Risk factor**	**Death in the ICU**			**Discharge from ICU**		
	
	**CSHR**	**95% CI**	**p Value**	**CSHR**	**95% CI**	**p Value**
Baseline						
Age (continuous variable)	1.02	1.01 to 1.03	< 0.01	1.00	1.00 to 1.01	0.01
Female gender	0.83	0.63 to 1.11	0.21	0.92	0.83 to 1.03	0.14
SAPS II (continuous variable)	1.02	1.01 to 1.03	< 0.01	0.98	0.98 to 0.98	< 0.01
Intubation on admission	0.83	0.69 to 1.00	0.06	1.14	1.04 to 1.24	< 0.01
Pneumonia on admission	0.72	0.47 to 1.10	0.13	0.61	0.51 to 0.72	< 0.01
LRT on admission	0.53	0.13 to 2.11	0.37	0.70	0.32 to 1.54	0.37
Urinary tract infection on admission	0.97	0.49 to 1.92	0.92	0.77	0.51 to 1.16	0.22
Other infections on admission	1.44	0.97 to 2.15	0.07	0.60	0.47 to 0.76	< 0.01
Hospitalization before admission	1.08	0.74 to 1.57	0.69	0.89	0.79 to 1.01	0.06
Surgical patients	0.56	0.34 to 0.93	0.03	1.02	0.86 to 1.20	0.83
Elective surgery before admission	0.43	0.31 to 0.58	< 0.01	0.56	0.50 to 0.63	< 0.01
Emergency surgery before admission	0.98	0.67 to 1.43	0.91	1.11	0.97 to 1.27	0.14
Cardial/pulmonary underlying disease	0.81	0.56 to 1.17	0.26	0.89	0.77 to 1.03	0.11
Neurological underlying disease	0.87	0.55 to 1.39	0.57	1.01	0.87 to 1.18	0.89
Metabolic/renal underlying disease	1.22	0.81 to 1.82	0.34	0.79	0.63 to 0.99	0.04
Other underlying disease	1.66	1.12 to 2.44	0.01	0.96	0.77 to 1.19	0.70
Time-dependent						
Ventilation	1.78	0.99 to 3.20	0.05	0.45	0.38 to 0.53	< 0.01
Chest drainage	0.99	0.70 to 1.41	0.97	0.71	0.62 to 0.82	< 0.01
Colostomy	0.96	0.26 to 3.61	0.95	0.59	0.23 to 1.53	0.28
Enterostomy	0.77	0.15 to 4.02	0.76	2.06	0.74 to 5.74	0.16
Jejunostomy	1.53	0.37 to 6.23	0.56	0.28	0.11 to 0.69	0.01
Nasogastric tube	0.82	0.45 to 1.50	0.52	0.89	0.76 to 1.04	0.14
Urinary catheter	0.74	0.43 to 1.27	0.27	0.78	0.66 to 0.94	0.01
Nosocomial pneumonia	0.87	0.56 to 1.36	0.55	0.59	0.49 to 0.71	< 0.01
Nosocomial blood stream infection	0.77	0.31 to 1.90	0.57	0.90	0.65 to 1.23	0.50
Nosocomial LRT	1.24	0.66 to 2.30	0.50	0.56	0.56 to 0.80	< 0.01

CSHR, cause-specific hazard ratio; LRT, lower respiratory tract infection (other than pneumonia); SAPS, simplified acute physiology score.

### Cumulative incidence functions (model 2)

**Figure 3:**
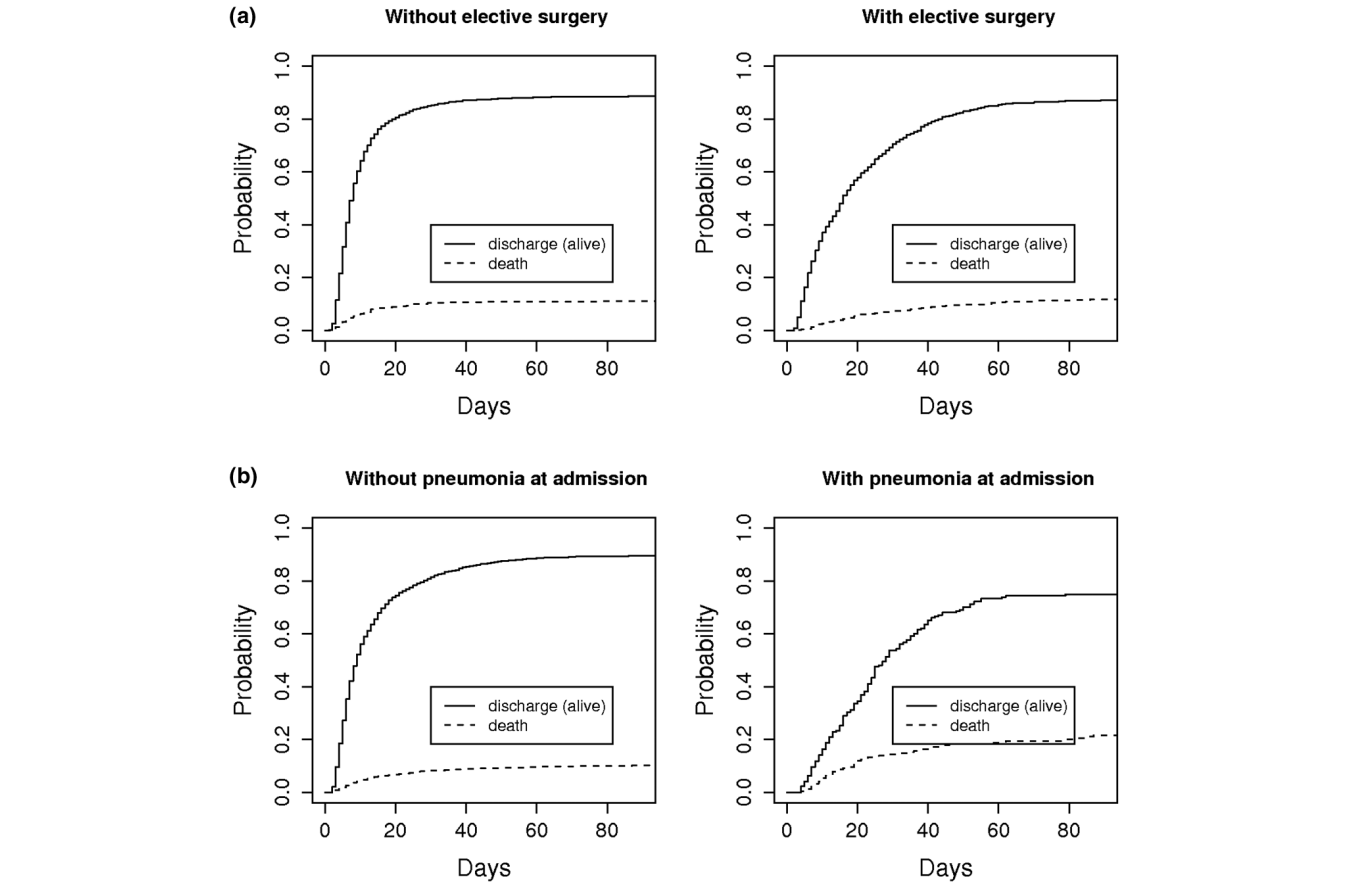
Cumulative incidence function for death and discharge (model 2). **(a) **In the two upper figures the risk factor 'elective surgery' is considered. **(b) **In the two lower figures the risk factor 'pneumonia on admission' is considered.

Although patients with an elective surgery had a lower cause-specific risk of death (CSHR = 0.43), they tended to stay longer in the ICU compared to those patients without an elective surgery (CSHR = 0.56). This effect can also be seen in Figure 3a: the cumulative incidences of both endpoints start at a later time point for patients with elective surgery.

Patients with pneumonia on admission stay longer in the ICU (CSHR = 0.61); the CSHR for death was not significant. However, that also means that patients with pneumonia on admission die more frequently. This effect can be viewed in Figure 3b: the cause-specific risk of death decreased for patients with pneumonia on admission at the beginning of their ICU stay, but increased if they stay longer; the curves intersect.

### Correlations among risk factors

The following time-dependent risk factors were highly correlated among each other: colostomy, enterostomy and jejunostomy (absolute values range between 0.6 to 0.9). There was a low correlation of the baseline risk factor 'intubated on admission' and the SAPS II score (0.5). All other correlation coefficients ranged between -0.4 and 0.4.

## Discussion

Many patient characteristics and significant risk factors for ventilator-associated pneumonia have been published. These include age, male gender, hospitalization prior to ICU admission, length of ICU stay, treatment in large hospitals, a low Glasgow Coma Scale (GCS), a poor Acute Physiology and Chronic Health Evaluation (APACHE) II or SAPS II score, respiratory failure, congestive heart failure, acute renal failure and dialysis, bronchoscopy, tracheotomy, re-intubation, duration of mechanical ventilation, detection of certain multi drug resistant pathogens, use of central vein catheters, bacteraemia, enteral feeding, and application of sucralfat or corticosteroids, [4,15-24].

However, in most of these studies the time-dependent issue of nosocomial infections was ignored, that is, the time-dependent exposure was analysed as being known at time origin. This results in time-dependent bias [25]. In addition, competing events such as discharge or death were not explicitly modelled. Recently, Resche-Rigon and co-authors point out that ICU discharge should be considered a competing event, when estimating the mortality of ICU patients [26]. In this context, Schoenfeld argued that one should better focus on whether patients die rather then when they die, and therefore mortality should be analysed as a binary variable (30-day mortality) using a logistic regression [27]. But that means that the time-dependent nature of nosocomial infections is ignored and it is impossible to consider time-dependent risk factors as for example, ventilation. In the present paper we applied multi-state models in order to accurately take these two important issues (that is, time-dependent risk-factors and competing events) into account.

The competing risks situation at hand, however, requires careful interpretation of the results: for example, in model 2 we find that pneumonia on admission has a (non-significant reducing) effect on the cause-specific hazard ratio of death, and an even more reducing (and significant) effect on the CSHR of discharge. This suggests that pneumonia on admission prolongs ICU stay; however, as the death hazard is not reduced as much as the discharge hazard is, there will eventually be more patients who are deceased [24]. Thus, the competing risks model explains how pneumonia on admission contributes to mortality: pneumonia on admission prolongs ICU stay; each day, such a patient is again exposed to the (not significantly altered) risk of dying. As a consequence, there will be more patients with pneumonia on admission, who stay longer and die in the ICU.

In this study, we could show that elective surgery increases the CSHR for nosocomial pneumonia (model 1). Although nosocomial pneumonia is a risk factor for death, patients with elective surgery have a lower cause-specific risk of dying (model 2). However, these patients stay longer in the ICU. There are two possible explanations for this: firstly, there is an effect independent of whether they acquire NP during their ICU stay, and secondly via a nosocomial pneumonia which extends their ICU stay as well.

Our data from a competing risk model 1 confirmed mechanical ventilation as the key risk factor for the development of NP, with an increase in the CSHR of 5.90 (Table 2); this effect is accentuated by the parallel competing risks analysis of CSHR for direct discharge, which is significantly reduced by mechanical ventilation. Additional significant factors in our study were some form of surgery prior to ICU stay and the use of a nasogastric tube, though as a limitation it should be remembered that we did not consider all of the above-mentioned factors from previous works. Patients with diagnosed pneumonia on admission were much less likely to develop NP (CSHR = 0.02; Table 2). Our interpretation of this is that very few patients resolve from the initial pneumonia, thus they cannot acquire an additional NP afterwards.

There is little doubt that the acquisition of NP increases the length of ICU stay and the overall health care costs [18,28]. However it is controversial whether NP also influences ICU mortality. Some studies found an increase in mortality due to NP, while other did not or found an increase for certain pathogens only [24]. When comparing and evaluating these findings the possibility of publication bias should be kept in mind. It is less likely that studies without a significant increase in mortality will get published. None of the studies carried out previously have ever used a model of time-dependent variables to address the question of the mortality attributable to NP. Our competing risk model 2 did not show an increase of the CSHR for a fatal outcome after NP (CSHR = 0.87; p = 0.55; Table 3). However, as stated above, patients with NP require longer treatment in the ICU on average. This was confirmed by our findings (CSHR for discharge = 0.59; p < 0.01; Table 3). As a consequence patients with NP are exposed to the (not significantly altered) risk of dying in the ICU for a longer time period compared to patients without NP. As a result of this, more patients will die after NP. This is a typical competing risks phenomenon, which is discussed in detail by Beyersmann *et al*. [29].

## Conclusion

More studies using competing risk models should be carried out to re-evaluate the impact of risk factors (especially time-dependent variables) on the occurrence of nosocomial infections and patient outcomes thereafter.

## Key messages

Nosocomial infections are time-dependent risk factors and should be analysed as such.

Ignoring the time-dependency of nosocomial infections leads to biased conclusions.

If the time to acquisition of a nosocomial infection is of interest, discharge/death is a competing event.

Whenever the length of ICU stay is of interest, death in the ICU is a competing event.

Only appropriate time-to-event analysis methods such as multi-state models can take the time-dependency of risk factors and competing events into account.

## Abbreviations

CDC = Centers for Disease Control and Prevention; CSHR = cause-specific hazard ratio; ICU = intensive care unit; KISS = German Nosocomial Infection Surveillance System; LRT = lower respiratory tract; NP = nosocomial pneumonia; SAPS = simplified acute physiology score.

## Competing interests

The authors declare that they have no competing interests.

## Authors' contributions

HG and PG initiated the SIR-3 study. MB created the database and online platform for the KISS system. SB and CG participated in collecting of the data. MW, JB and MS participated in the statistical analysis of the data. RPV, PG and HR participated in interpreting the data and drafting of the manuscript. All authors read and approved the final manuscript.

## Supplementary Material

Additional file 1 Additional file 1 contains information on the required data format and SAS and R calculations for cause-specific hazard ratios in a competing risks analysis with time-dependent covariates represented. Click here for file
