# Supplementary material for: Risk factors for the development of nosocomial pneumonia and mortality on intensive care units: application of competing risks models
Source: Crit Care. 2008 Apr 2;12(2):R44. doi: 10.1186/cc6852 (PMC2447589; doi:10.1186/cc6852)
Supplement: Additional file 1 — contains information on the required data format and SAS and R calculations for cause-specific hazard ratios in a competing risks analysis with time-dependent covariates represented. [file cc6852-S1.pdf]

SAS and R calculations for cause-specific hazard ratios in a competing risks analysis with time-dependent covariates

Martin Wolkewitz , Ralf-Peter Vonberg, Hajo Grundmann, Jan Beyersmann, Petra Gastmeier, Sina Bärwolff, Christine Geffers, Michael Behnke, Henning Rüden, Martin Schumacher

Abstract

In this supplement, we demonstrate how to calculate the cause-specific hazard ratios (CSHR) using the software SAS (SAS Institute, Inc., Cary, North Carolina) and the free software R (1). Special focus is given on the counting process format which is a convenient data representation of time-dependent covariates. Motivated from hospital epidemiology, a typical situation in which the impact of a time-dependent exposure (nosocomial pneumonia) on two competing endpoints (death and discharge) is exemplarily considered.

Introduction

Typically, survival analysis models the time to event; if there are several events present, a competing risks model is needed to take multiple types of events into account. The standard approach is modeling the cause-specific hazard function (2). We present the SAS- and R-code for epidemiologists who are basically familiar with the methodological background of competing risks theory; we refer to the tutorial by Putter et al. (2). The inclusion of binary time-dependent covariates is explicitely explained.

Non-reversible binary time-dependent covariates

The simplest time-dependent covariate is a non-reversible binary variable, whose values changes from 0 to 1 at the time of occurence of the intermediate event. It is defined as follows:

$$Z_i(t) = \begin{cases} 0 \text{ if } t \leq \text{time of occurence of the intermediate event (unexposed)} \\ 1 \text{ if } t > \text{time of occurence of the intermediate event (exposed)} \end{cases}$$

Such a variable occurs very frequently, e.g. as an exposure in epidemiology, and describes a subject who enters the study unexposed, gets exposed at time t and stays in this exposed status until the study endpoint. In fact, there are various types of time-dependent covariates. Here, we focus on this special type and refer the interested reader to (3).

Regression on cause-specific hazards

The cause-specific hazard of cause k for a subject with a covariate vector $Z(t) = (Z_1(t),...,Z_p(t))$, which may contain time-dependent as well as time-independent covariates, is modeled as

$$\lambda_k(t \mid Z(t)) = \lambda_{k,0}(t)\exp(\beta_1 Z_1(t) + ... + \beta_p Z_p(t))$$

where $\lambda_{k,0}(t)$ is the baseline cause-specific hazard of cause k and β_k are the regression coefficients that represents the covariate effects on cause k.

Counting process format for time-dependent covariates

This style can handle time-dependent covariates as well as left-truncation and right-censoring with controlling the risk set. For each patient, one row represents a time interval (start,stop], a status indicator and the values of the covariates. Here, we only consider one time-dependent covariate (e.g. NP=nosocomial pneumonia, definition as above). For each patient, there are two possible outcomes: death or discharge. The status_ variable indicates the status on the ‚stop'-day: status_=0 indicates no endpoint, status_=1 death and status_=2 indicates discharge. The data might look as follows:

**patient**	**start**	**stop**	**status_**	**NP**
1	0	4	0	0
1	4	10	1	1
2	0	12	0	0
2	12	15	2	1
3	0	20	2	0
4	0	5	0	0
5	0	7	0	0
5	7	13	0	1

In our hypothetical example, patient 1 enters the study free of NP, acquires NP on day 4 and dies on day 10. Patient 2 acquires NP on day 12 and is discharged on day 15. Patient 3 stays free of NP until his discharge on day 20. Patient 4 is administratively censored on day 5 and did not acquire NP until this day whereas patient 5 acquires NP on day 7 before being administratively censored on day 13.

Blowing up the data to the long format

In order to fit one stratified analysis according to the competing endpoints, the data requires the long format. We include the variables ‚endpoint' and ‚status' and create two new dummy variables NP_death and NP_discharge (type-specific covariates).

patient	start	stop	status	endpoint	NP_death	NP_discharge
1	0	4	0	death	0	0
1	4	10	1	death	1	0
1	0	4	0	discharge	0	0
1	4	10	0	discharge	0	1
2	0	12	0	death	0	0
2	12	15	0	death	1	0
2	0	12	0	discharge	0	0
2	12	15	1	discharge	0	1
3	0	20	0	death	0	0
3	0	20	1	discharge	0	0
4	0	5	0	death	0	0
4	0	5	0	discharge	0	0
5	0	7	0	death	0	0
5	7	13	0	death	1	0
5	0	7	0	discharge	0	0
5	7	13	0	discharge	0	1

SAS or R codes to yield CSHR

For fitting a stratified Cox model assuming proportional hazards the PHREG procedure in SAS (4,5) or, alternatively, the *coxph* function from the *survival* R-package may be used.

Software	CSHR for death
SAS	proc phreg covsandwich(aggregate) covm;<br>model (start,stop)*status(0) = NP_death NP_discharge;<br>strata endpoint;<br>id patient;<br>run;
R	> coxph(Surv(start,stop,status = =1) ~ NP_death + NP_discharge + strata(endpoint) + cluster(patient))

Please note, that status(0) indicates to SAS that 0 is technically considered censoring and the value 1 is the event of interest. In R, status = = 1 indicates the endpoint and the other possible values as censored.

Robust / Sandwich variance

Data contain several records per patient, thus robust estimates of standard errors might be one way to take this correlation into account. This can be done by specifying the SAS-option ‚covsandwich(aggregate) covm' in the PHREG procedure in combination with the ID statement. In R one has to add ‚cluster(patient)' to yield robust estimates.

Remarks

Often, the data requires careful preparation in order to receive the counting process format. We strongly recommend to check the dataset before fitting the model. Several time-dependent as well as time-independent covariates may be described in this format.

Reference

1) Ihaka R, Gentleman R: **R: A Language for Data Analysis and Graphics**, Journal of Computational and Graphical Statistics, 1996, vol. 5 (3), 299-314

2) Putter H, Fiocco M, Geskus RB: **Tutorial in biostatistics: competing risks and multi-state models.** *Stat Med* 2007, 26:2389-2430.

3) Klein JP, Moeschberger L: **Survival analysis: techniques for censored and truncated data**, 2nd ed., 2003 Springer

4) Allison PD: **Survival Analysis Using the SAS® System: A Practical Guide**, Cary, NC: *SAS Institute Inc.*, 1995. 292 pp.

5) Ake CF, Carpenter AL: **Extending the Use of PROC PHREG in Survival Analysis**, *SAS Conference Proceedings*: WUSS 2003, San Francisco, California
